# Silica-based cationic bilayers as immunoadjuvants

**DOI:** 10.1186/1472-6750-9-5

**Published:** 2009-01-19

**Authors:** Nilton Lincopan, Mariana RA Santana, Eliana Faquim-Mauro, Maria Helena B da Costa, Ana M Carmona-Ribeiro

**Affiliations:** 1 Departamento de Bioquímica, Instituto de Química, Universidade de São Paulo, São Paulo, Caixa Postal 26077, São Paulo-SP, Brazil; 2 Instituto Butantan, Av. Vital Brasil 1500, Butantan 05503-900, Brazil

## Abstract

**Background:**

Silica particles cationized by dioctadecyldimethylammonium bromide (DODAB) bilayer were previously described. This work shows the efficiency of these particulates for antigen adsorption and presentation to the immune system and proves the concept that silica-based cationic bilayers exhibit better performance than alum regarding colloid stability and cellular immune responses for vaccine design.

**Results:**

Firstly, the silica/DODAB assembly was characterized at 1 mM NaCl, pH 6.3 or 5 mM Tris.HCl, pH 7.4 and 0.1 mg/ml silica over a range of DODAB concentrations (0.001–1 mM) by means of dynamic light scattering for particle sizing and zeta-potential analysis. 0.05 mM DODAB is enough to produce cationic bilayer-covered particles with good colloid stability. Secondly, conditions for maximal adsorption of bovine serum albumin (BSA) or a recombinant, heat-shock protein from *Mycobacterium leprae *(18 kDa-hsp) onto DODAB-covered or onto bare silica were determined. At maximal antigen adsorption, cellular immune responses *in vivo *from delayed-type hypersensitivity reactions determined by foot-pad swelling tests (DTH) and cytokines analysis evidenced the superior performance of the silica/DODAB adjuvant as compared to alum or antigens alone whereas humoral response from IgG in serum was equal to the one elicited by alum as adjuvant.

**Conclusion:**

Cationized silica is a biocompatible, inexpensive, easily prepared and possibly general immunoadjuvant for antigen presentation which displays higher colloid stability than alum, better performance regarding cellular immune responses and employs very low, micromolar doses of cationic and toxic synthetic lipid.

## Background

Over the last two decades novel assemblies obtained from particles and lipids have been introduced as important tools to novel applications in drug and vaccine delivery [1-4]. Particulates such as silica, latex or hydrophobic drugs have been coated by lipids and successfully employed in biomolecular recognition [5,6] drug delivery [7,8] and antigen presentation [9,10]. The systematic and quantitative evaluation of particle-lipid interaction has been realized by means of adsorption isotherms of lipids on particles, effects of lipids on particle size and zeta-potential from dynamic light scattering methods and determination of colloid stability from turbidity kinetics or particle sedimentation over time [1-10]. Cationic lipids, in particular, are especially interesting to cover particles, since cationic particles may electrostatically combine with a vast variety of oppositely charged biomolecules, cells or other biological structures. Cationization, in general, has often been explored as a convenient approach to target active biomolecules into cells [11].

The control of lipid assembly on particles turned out to be dependent on properties of the intervening medium, eg ionic strength, and on the proportion of surface areas for bilayer vesicles and particles in dispersion [12-15]. From equivalence of total surface areas for particles and cationic lipid bilayers, over a range of low ionic strength, a good colloid stability was reported for the bilayer-covered cationic particles [3,4,12,15].

In this work, the interaction between silica previously coated with cationic bilayers of dioctadecyldimethylammonium bromide (DODAB) [15] and the model protein bovine serum albumin (BSA) is investigated aiming at antigen presentation to the immune system by silica-based cationized particles. BSA choice was due to various reasons: its extensively studied adsorption behaviour at interfaces [16,17]; its utility to prevent nonspecific binding in biosensing and proteomics applications [17-19], its conformational adaptability as a "soft" globular protein [20], its thoroughly investigated adsorption onto hydrophobic or hydrophilic particles sometimes resulting in exchange between the adsorbed and dissolved states [21-24] and its substantial adsorption onto cationic and large DODAB vesicles [25].

The 18 kDa-hsp protein belongs to a conserved protein family of *M. leprae *heat-shock proteins that display pronounced immunogenicity and are considered important targets of the immunoresponse to mycobacteria and, as such, relevant to subunit vaccine design. Peripheral blood mononuclear cells and T-cell lines from *M. leprae *vaccinated subjects proliferated in response to this protein [26]. Furthermore, overexpression and scaling-up of 18 kDa-hsp production in *Saccharomyces cerevisae *has already been described so that this protein is available in sufficient amount for a complete physico-chemical study of the adjuvant-antigen interaction [27-29].

The DODAB cationic lipid and its assemblies in water dispersion have been established as effective immunoadjuvants able to stimulate dendritic cells and often employed to present antigens [29-34]. Silica particles are biocompatible, represent a reference adsorbent, offer a chemically well defined surface and are widely used as a chromatographic stationary phase [34,35].

We have recently combined the typical property of particles that stimulate dendritic cells uptake with the adjuvant effect of DODAB by using supported DODAB bilayers on latex to present antigens [9]. Here we take advantage of the biocompatible character of silica [35,36] to produce DODAB-covered silica particles for further immobilization and presentation of two different model antigens: BSA and 18 kDa-hsp protein.

## Results and discussion

### Coverage of silica particles with a cationic bilayer and BSA adsorption

Charge density on silica particles increases with pH and ionic strength [37] so that electrostatic attraction between DODAB bilayer and silica is substantial over the 1–10 mM range of monovalent salt concentration, and leads to DODAB bilayer deposition onto particles [15]. One should notice that poor or none DODAB adsorption on silica was previously reported for pure water as intervening media [13-15,38]. Therefore, the experiments in this work were designed either at 1 or at 5 mM monovalent salt.

The DODAB bilayer in closed vesicles is in the rigid gel state at room temperature. This represents an important limitation regarding deposition of bilayers onto particles hampering the occurrence of vesicle disruption which is essential for bilayer deposition. We have previously shown that closed vesicles with bilayers in the gel state do not disrupt upon contact with silica particles [38], thus, in this work, bilayer deposition on silica is obtained by employing disrupted vesicles or bilayer fragments.

**Figure 1:**
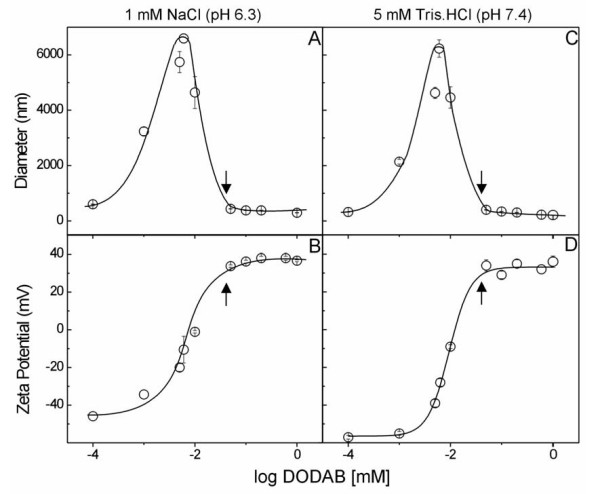
**The concentration of cationic lipid DODAB required to cover silica particles with a cationic lipid bilayer is 0.05 mM**. Effect of DODAB concentration on zeta-average diameter (A, C) and zeta-potential (B, D) of SiO_2 _particles at 0.1 mg silica/ml, 25°C, in 1 mM NaCl, pH 6.3 (A, B) or 5 mM Tris.HCl, pH 7.4 (C, D). Cationic bilayer fragments were adsorbed onto 0.1 mg/ml silica particles (AEROSIL OX-50). Measurements were performed after 1 h interaction. The arrows indicate 0.05 mM DODAB as the concentration of cationic lipid that is required to attain maximal positive zeta-potential and minimal sizes for particles in dispersion.

Both at 1 mM NaCl, pH 6.3, and at 5.0 mM Tris.HCl, pH 7.4, the dependence of size and zeta-potential of silica particles on DODAB concentration shows size minimization and zeta-potential maximization at and above 0.050 mM DODAB (Figure 1). Therefore, 0.050 mM DODAB was the final concentration selected to cover silica particles at 0.1 mg/ml with one DODAB bilayer. Theoretically, assuming 0.6 nm^2 ^as the DODAB area per molecule at the air-water interface and 26–50 m^2^/g as silica specific surface area, the DODAB concentration required to cover silica particles at 0.1 mg/ml with one bilayer would be 0.014–0.028 mM DODAB, in reasonable agreement with the 0.050 mM DODAB experimentally determined for minimization of particle size and maximal zeta-potential (Figure 1). The bell-shaped dependence of zeta-average diameter of particles as a function of the logarithm of DODAB concentration shows the occurrence of two regions of high colloidal stability identified by low sizes and large zeta-potentials in modulus: one at negative and the other at positive zeta-potentials both at mean sizes similar to the one of bare silica and over a range of low and high DODAB concentrations (Figure 1).

The stability of the silica/DODAB system was sistematically described in a previous work [15]. The adjuvant system is very reproducible yielding always the same mean Dz and zeta-potential for the same final silica and DODAB concentrations. However, upon antigen addition, zeta-potential may decrease to values close to zero so that colloid stability will be low.

Table 1 shows the detailed physical description of silica, DODAB bilayer fragments (BF), silica/DODAB and silica/DODAB/BSA regarding mean particle size, zeta-potential and polydispersity. In agreement with Tadros and Lyklema data [37], silica in 1 mM NaCl at pH 6.3 was less charged (zeta-potential of -40 mV) than at pH 7.4 (zeta-potential of -47 mV). One should notice the aggregated state of BSA in solution and the large polydispersity of these aggregates at 0.5 mg/ml in 1 mM NaCl and pH 6.3 or in 5 mM Tris.HCl, pH 7.4 (Table 1). Consistently, at pH 7.4, the protein aggregates display a more negative zeta-potential than the one at pH 6.3, as expected from BSA isoelectric point at pH 5.0–5.5. When the protein was added to silica/DODAB particles to a final [BSA] of 0.025 mg/ml, different assemblies were obtained at pH 6.3 and 7.4 (Table 1): the larger electrostatic attraction at pH 7.4 drove BSA onto particles to yield a slightly negatively charged particle whereas, at pH 6.3, the moderate electrostatic attraction, moderately drove BSA onto particles yielding a still positively charged particle with 24 mV of zeta-potential (Table 1). The design chosen for evaluation of BSA presentation to the immune system was the one in 5 mM Tris.HCl, pH 7.4 yielding mean diameters of ca. 800 nm. All other results in Table 1, consistently agreed with previous data published by our group.

**Table 1:** Physical properties of SiO_2_, DODAB BF dispersion, BSA, 18 kDa-hsp *leprae *and their mixtures in 1 mM NaCl (pH 6.3) or in 5 mM TrisHCl (pH 7.4)

**Dispersion**	**SiO_**2**_** **(mg/ml)**	**DODAB** **(mM)**	**Protein** **(mg/ml)**	**Mean Diameter** **(nm)**	**Zeta Potencial** **(mV)**	**Polydispersity** **(Index)**
SiO_2_^(*i*)^	0.1	-	-	328 ± 7	-40 ± 2	0.240 ± 0.01
DODAB^(*i*)^	-	1.0	-	67 ± 1	41 ± 3	0.120 ± 0.04
BSA^(*i*)^	-	-	0.5	25 ± 4	-28 ± 1	0.450 ± 0.09
DODAB/BSA^(*i*)^	-	0.1	0.500	72 ± 1	3 ± 1	0.245 ± 0.01
SiO_2_/DODAB^(*i*)^	0.1	0.05	-	304 ± 2	32 ± 1	0.260 ± 0.02
SiO_2_/DODAB/BSA^(*i*)^	0.1	0.05	0.025	324 ± 8	24 ± 1	0.301 ± 0.01
SiO_2_^(*ii*)^	0.1	-	-	294 ± 2	-47 ± 2	0.180 ± 0.02
DODAB^(*ii*)^	-	1.0	1.0	74 ± 1	33 ± 5	0.270 ± 0.04
BSA^(*ii*)^	-	-	0.5	24 ± 1	-58 ± 18	0.397 ± 0.01
SiO_2_/DODAB^(*ii*)^	0.1	0.05	-	373 ± 7	33 ± 2	0.260 ± 0.02
SiO_2_/DODAB/BSA^(*ii*)^	0.1	0.05	0.025	851 ± 36	-1.3 ± 4	0.396 ± 0.03
18 kDa-hsp *M. leprae*^(*i*)^	-	-	0.5	732 ± 188	-29 ± 2	0.35 ± 0.05
DODAB/18 kDa-hsp^(*i*)^	-	0.1	0.05	208 ± 8	39 ± 5	0.315 ± 0.01
18 kDa-hsp *M. leprae*^(*ii*)^	-	-	0.05	360 ± 16	-43 ± 18	0.180 ± 0.09
SiO_2_/DODAB/18 kDa-hsp^(*ii*)^	0.1	0.05	0.05	2132 ± 370	-2 ± 2	0.492 ± 0.04
Al(OH_3_)^(*i*)^	-	-	-	458 ± 3	28 ± 3	0.191 ± 0.04
Al(OH_3_)^(*ii*)^	-	-	-	702 ± 17	14 ± 2	0.317 ± 0.01
Al(OH_3_)/18 kDa-hsp^(*i*),(*iii*)^	-	-	0.05	4888 ± 665	-	0.272 ± 0.07
Al(OH_3_)/18 kDa-hsp^(*ii*),(*iii*)^	-	-	0.05	10607 ± 2998	-	0.480 ± 0.03

(i) 1 mM NaCl (pH 6.3). (ii) 5 mM Tris-HCl (pH 7.4). (iii) Al(OH_3_) at 0.1 mg/ml.

**Figure 2:**
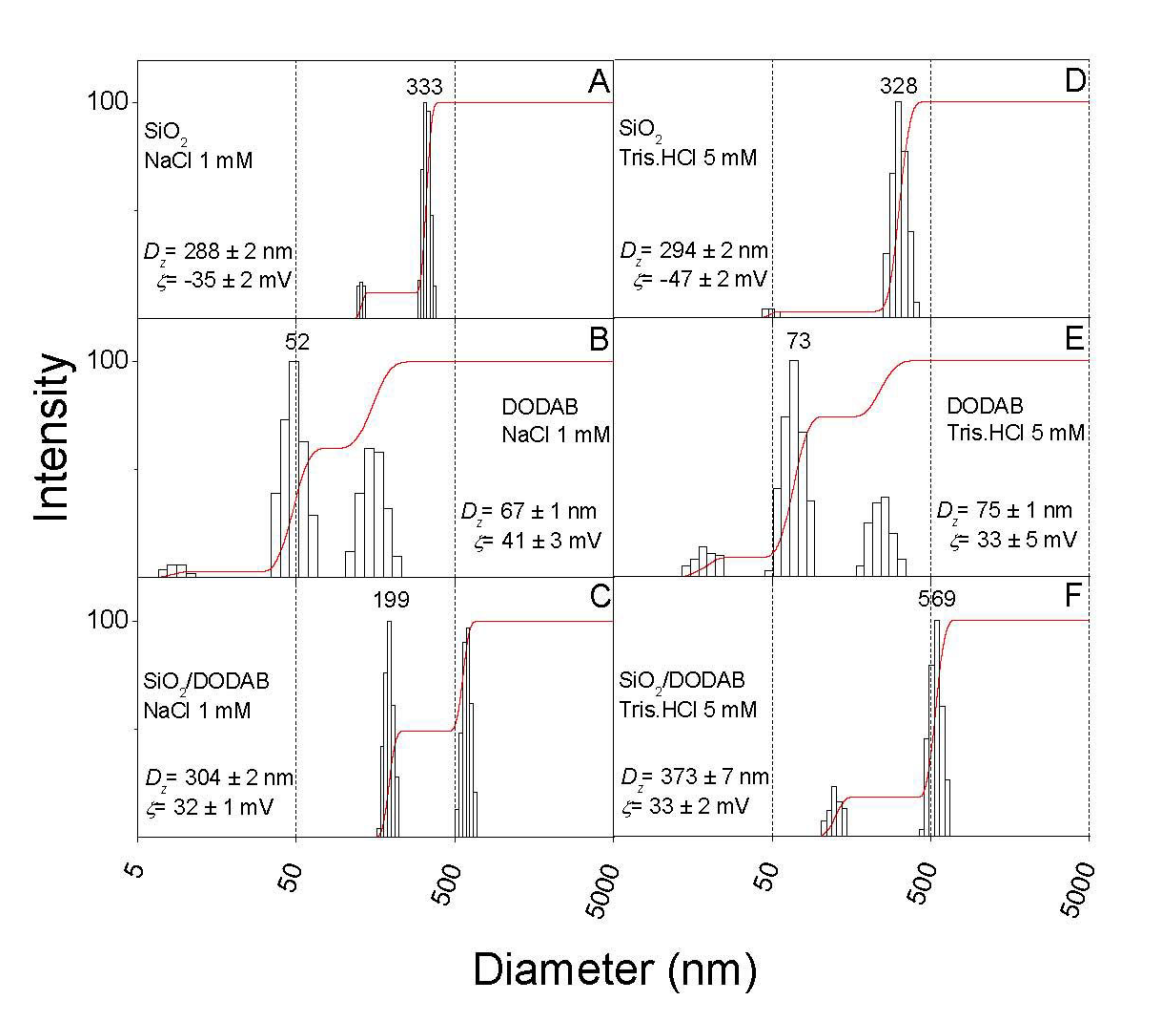
**Comparison of size distributions and zeta-potentials for silica and silica-based cationic bilayers**. Size distribution and zeta-potential (ζ) for 0.1 mg/ml silica, dispersion of 1 mM DODAB bilayer fragments and silica/DODAB particles prepared from 0.1 mg/ml silica and 0.05 mM DODAB. The intervening medium was 1 mM NaCl, pH 6.3 (A-C) or 5 mM Tris.HCl, pH 7.4 (D-F). Measurements were performed, after 1 h interaction. In each subfigure, mean zeta-potential and diameter ± SE are quoted.

**Figure 3:**
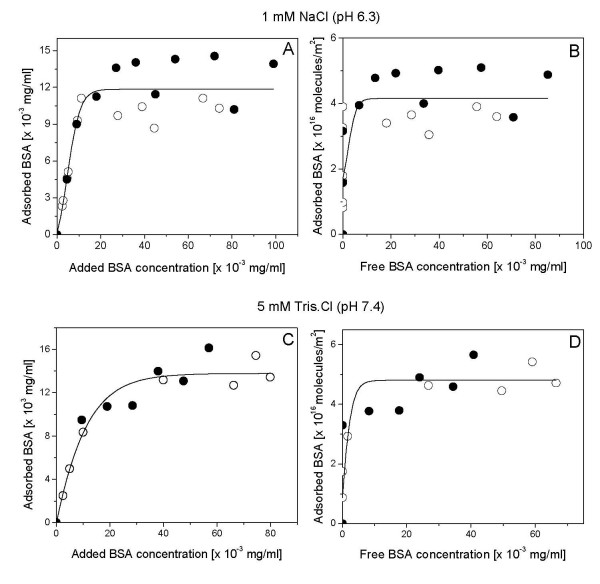
**Low levels of maximal BSA adsorption onto bare silica**. BSA adsorption isotherms onto bare SiO_2 _particles in two different media: 1 mM NaCl, pH 6.3 or 5 mM Tris.HCl, pH 7.4. Open and filled circles refer to two independent experiments with each mixture evaluated in duplicate. Interaction between silica particles and protein took place for 1 h at 25°C. Final silica concentration is 0.1 mg/ml. BSA adsorption was expressed either as adsorbed BSA concentration in mg/ml (A and C) or as number of BSA molecules adsorbed per m^2 ^silica (B and D).

**Figure 4:**
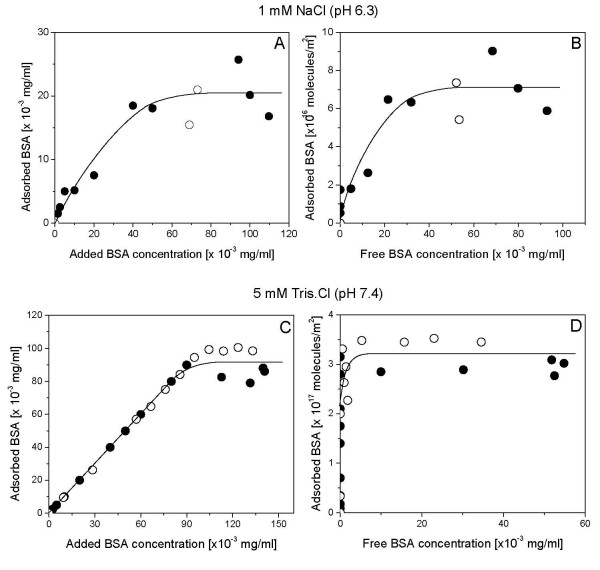
**High levels of maximal BSA adsorption onto silica-based cationic bilayers**. BSA adsorption isotherms onto silica/DODAB particles in two different media. 1 mM NaCl, pH 6.3 or 5 mM Tris.HCl, pH 7.4. Open and filled circles refer to two independent experiments with each mixture evaluated in duplicate. Interaction between silica/DODAB particles and protein took place for 1 h at 25°C. Final silica and DODAB concentrations are 0.1 mg/ml and 0.05 mM, respectively. BSA adsorption was expressed as adsorbed BSA concentration in mg/ml (A and C) or as number of BSA molecules adsorbed per m^2 ^silica (B and D).

Figure 2 shows size distributions for silica, DODAB BF and silica/DODAB for each dispersion both at 1 mM NaCl, pH 6.3 and at 5 mM Tris. HCl, pH 7.4. Under both experimental conditions, DODAB addition produced the silica-based positively charged particles with final size distributions similar to the ones of bare silica. Quantification of BSA amount adsorbed from solution onto bare silica (Figure 3) or onto silica/DODAB (Figure 4) in the two quoted intervening media was carried out over a range of added BSA concentrations (0–150 μg/ml). Given the langmuirian profile of the adsorption isotherms, we applied the Langmuir model in order to linearize adsorption isotherms and obtain adsorption parameters prone to be compared with each other for different intervening media and silica or silica/DODAB as adsorbates. Table 2 summarizes the BSA adsorption parameters such as maximal adsorption and affinity constant for adsorption onto bare silica or onto silica/DODAB particles in the two different intervening media. BSA adsorbs with the highest affinity onto bare silica, possibly driven by several hydrogen bonds between silanoils on particles and carbonyls on the protein, but maximal adsorption is at lowest indicating the largest area per BSA molecule occupied at maximal adsorption among those in all media and adsorbates tested (Table 2). Changing the medium to 5 mM Tris.HCl and pH 7.4, diminished BSA affinity for the negatively charged bare silica since electrostatic repulsion was increased by increasing negative charge on the protein though maximal adsorption remained approximately the same (Table 2). Offering the DODAB bilayer on silica to the protein improved BSA maximal adsorption for both intervening media. However, a large increase in the affinity constant such as the one obtained for BSA adsorption on silica/DODAB in Tris.HCl pH 7.4 (Table 2) may not be so desirable in terms of molecular order of BSA on the particle surface. While BSA appeared to be in a ordered end-on position occupying about 14 nm^2 ^at maximal adsorption (Table 2) under conditions of moderate electrostatic attraction between BSA and silica/DODAB (1 mM NaCl, pH 6.3) further increasing this attraction drove massive BSA adsorption onto the particle as detected from the small area occupied per protein on the silica/DODAB surface, about 3 nm^2 ^(Table 2). It was previously reported by us that BSA maximal adsorption onto supported DODAB bilayers deposited on polystyrene sulfate microspheres with 1 mM NaCl, pH 6.3 as the intervening medium was also consistent with deposition of an end-on BSA monomolecular layer [24]. This agreement for different supporting particles reconfirms not only DODAB bilayer deposition on silica but also suggests the preferential end-on mode of BSA adsorption on DODAB bilayer at pH 6.3 and 1 mM NaCl.

**Table 2:** Maximal adsorption, affinity constant (*K*) and area per adsorbed BSA molecule on silica (0.1 mg/ml) or on DODAB covered silica particles in two different media

**Particles**	**Maximal adsorption** **(molecules m^**-2**^)**	**Affinity Constant** **(*K*, M^**-1**^)**	**Area per adsorbed molecule** **(nm^**2**^)**
SiO_2_/BSA^(*i*)^	4.14 × 10^16^	1.19 × 10^9^	24.15
SiO_2_/BSA^(*ii*)^	4.75 × 10^16^	8.19 × 10^7^	21.05
SiO_2_/DODAB/BSA^(*i*)^	7.20 × 10^16^	5.18 × 10^7^	13.88
SiO_2_/DODAB/BSA^(*ii*)^	3.22 × 10^17^	8.98 × 10^8^	3.10
SiO_2_/18 kDa-hsp^(*ii*)^	1.12 × 10^17^	-	-
SiO_2_/DODAB/18 kDa-hsp^(*ii*)^	10.00 × 10^17^	-	-

(i) 1 mM NaCl (pH 6.3).

(ii) 5 mM Tris.HCl (pH 7.4).

Final DODAB concentration was 0.05 mM.

**Figure 5:**
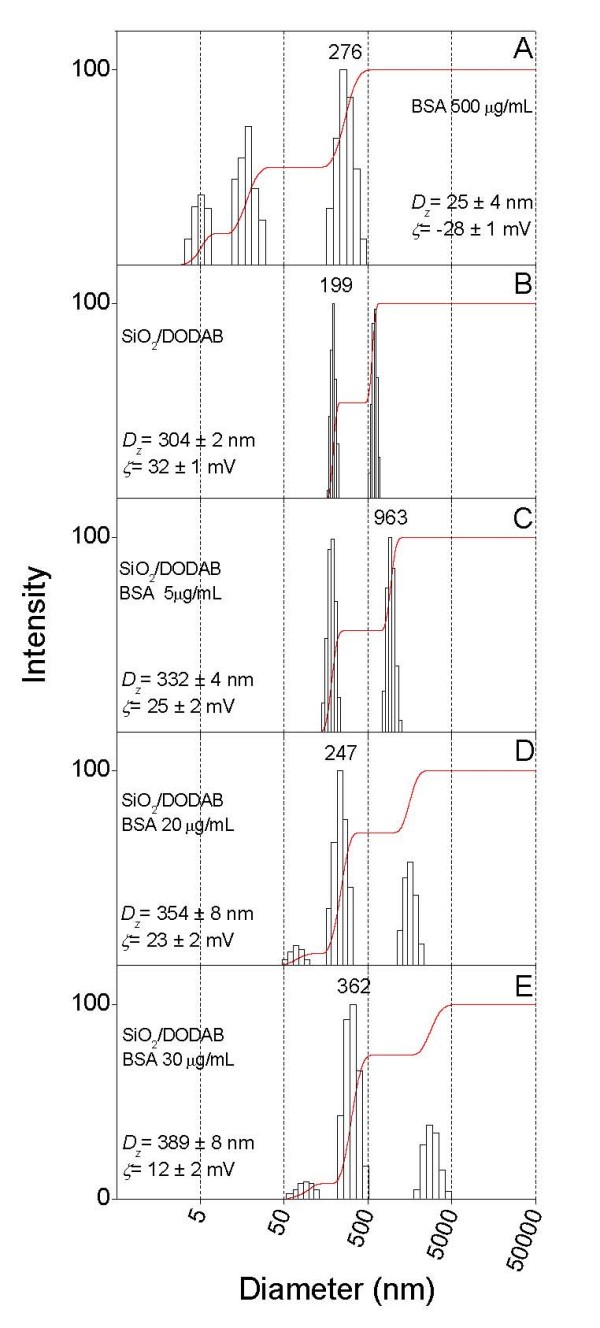
**BSA adsorption in 1 mM NaCl pH 6.3 affects silica/DODAB particle size and zeta-potential**. Size distribution and zeta-potential (ζ) for dispersions in 1 mM NaCl at pH 6.3. In (A), 500 μg/ml BSA. In (B), 0.1 mg/ml silica and 0.05 mM DODAB BF dispersion. From C to E, 0.1 mg/ml silica, 0.05 mM DODAB BF dispersion and BSA at 0.005 (C), 0.020 (D) and 0.030 mg/ml (E). Interaction between components in the mixtures took place over 1 h before measurements. In each subfigure, mean zeta-potential and diameter ± SE are quoted.

**Figure 6:**
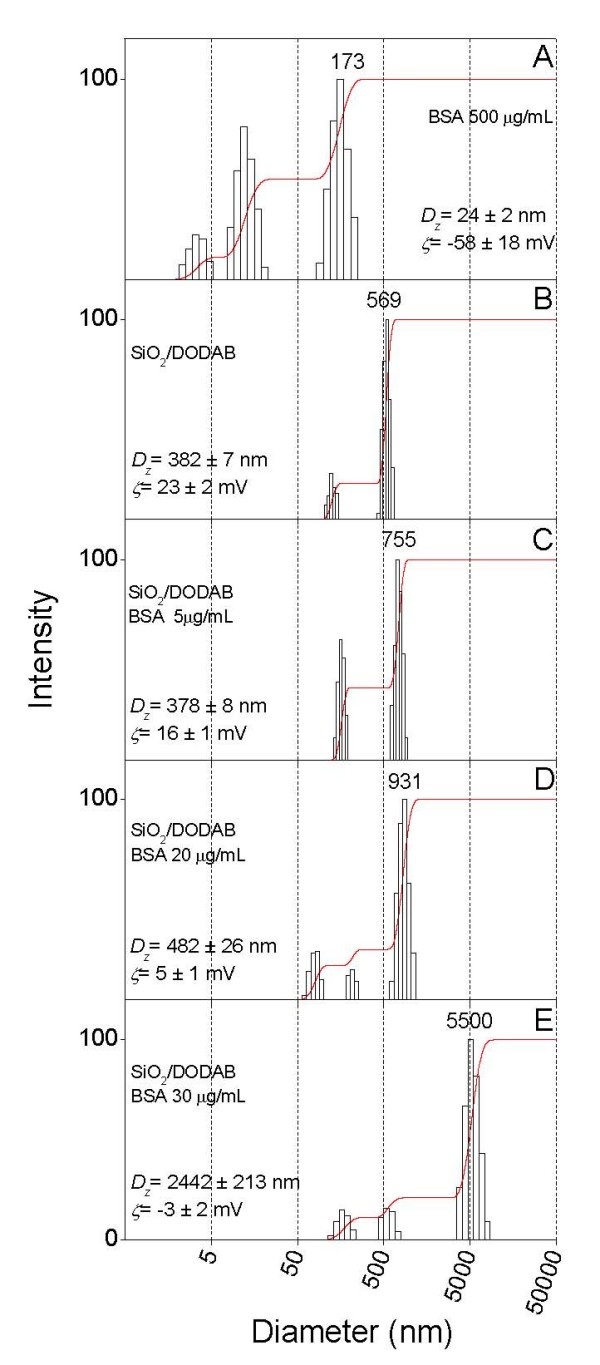
**BSA adsorption in 5 mM Tris.HCl, pH 7.4 affects silica/DODAB particle size and zeta-potential**. Size distribution and zeta-potential (ζ) for dispersions in 5 mM Tris.HCl, pH 7.4. In (A), 500 μg/ml BSA. In (B), 0.1 mg/ml silica and 0.05 mM DODAB BF dispersion. From C to E, 0.1 mg/ml silica, 0.05 mM DODAB BF dispersion and BSA at 0.005 (C), 0.02 (D) and 0.03 mg/ml (E). Interaction between components in the mixtures took place over 1 h before measurements. In each subfigure, mean zeta-potential and diameter ± SE are quoted.

Size distributions for BSA aggregates, silica/DODAB and silica/DODAB/BSA particles are on Figure 5 and Figure 6 for dispersions in 1 mM NaCl, pH 6.3 and in 5 mM Tris.HCl, pH 7.4, respectively. Interestingly enough, increasing BSA concentration from 5 up to 30 μg/ml, colloid stability rapidly decreased for the latter environment yielding aggregates with ca. 2.5 μm mean diameter and zeta-potentials close to zero (Figure 6E). The moderate electrostatic attraction in 1 mM NaCl, pH 6.3 produced higher colloid stability for silica/DODAB/BSA assemblies given their positive charge even at the largest BSA concentration (30 μg/ml) (Figure 5E). However, we privileged the largest adsorbed amount of BSA obtained in Tris.HCl, pH 7.4 for testing immune response.

### Silica-based cationic bilayers for induction of humoral immune response

**Figure 7:**
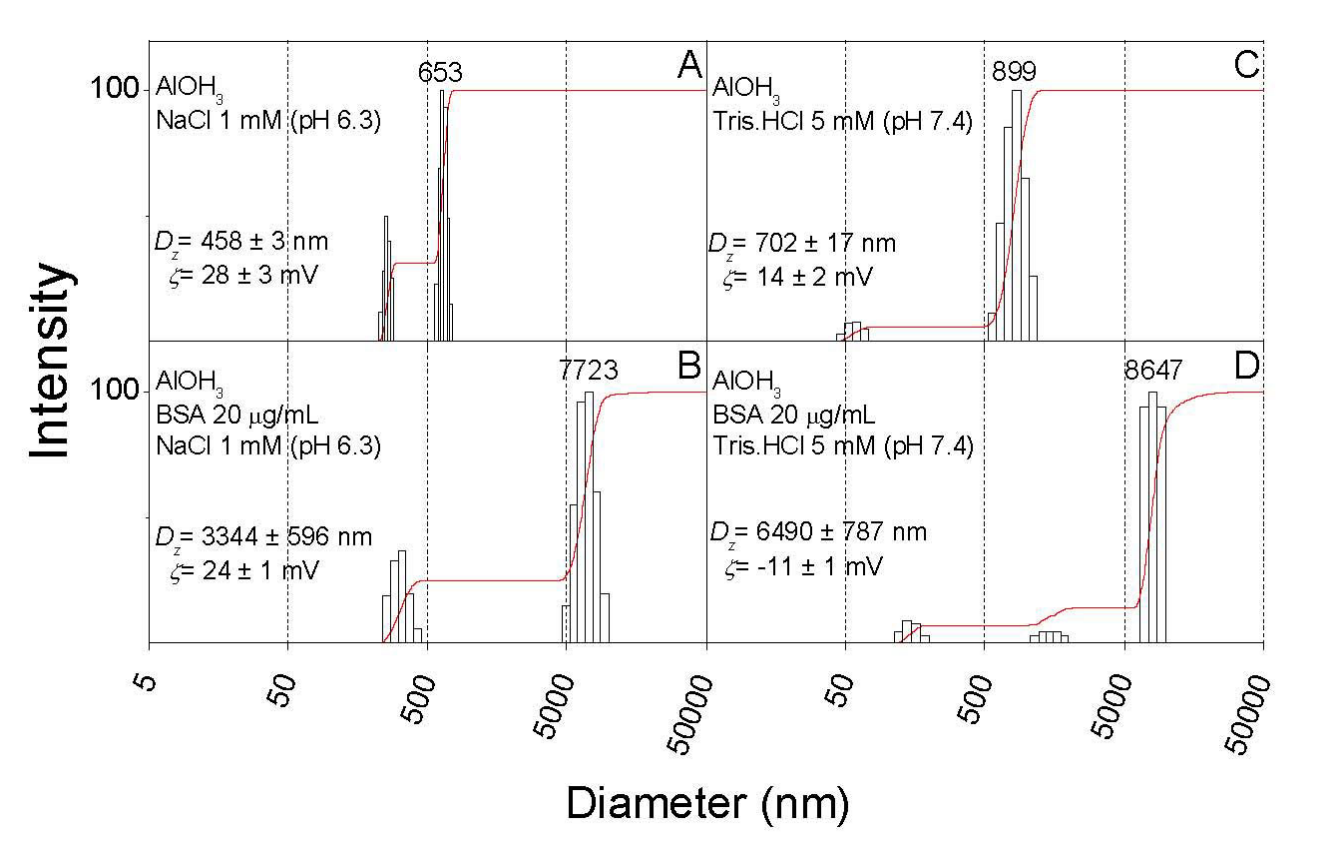
**BSA induces extensive alum aggregation strongly affecting its size distribution and zeta-potential**. Size distribution and zeta-potential (ζ) for Al(OH_3_) (A and C) or Al(OH_3_)/BSA dispersions (B and D) at 0.1 mg/ml Al(OH3), 1 mM NaCl, pH 6.3 (A and B) or 5 mM Tris.HCl, pH 7.4 (C and D).

Vaccines based on recombinant protein antigens require an adjuvant to elicit an immune response [39]. The most popular, widely used and the only currently FDA-approved adjuvant class in the USA is represented by aluminum salts [40]. Among these, aluminum hydroxide has a point of zero charge at pH 9–11 so that below this range, particles are positively charged [40]. For the sake of comparison, size distributions and zeta-potentials were obtained under similar conditions both for aluminum hydroxide and for silica/DODAB particles (Figure 7). Aluminum hydroxide presents a low colloid stability in the presence of BSA as depicted from the large mean particle sizes obtained both for positively or negatively charged final assemblies with BSA. The comparison with silica/DODAB data in Figures 5 and 6, clearly points out the adequacy of the silica-based supported cationic bilayers as more stable adjuvants from the point of view of colloid stability.

**Figure 8:**
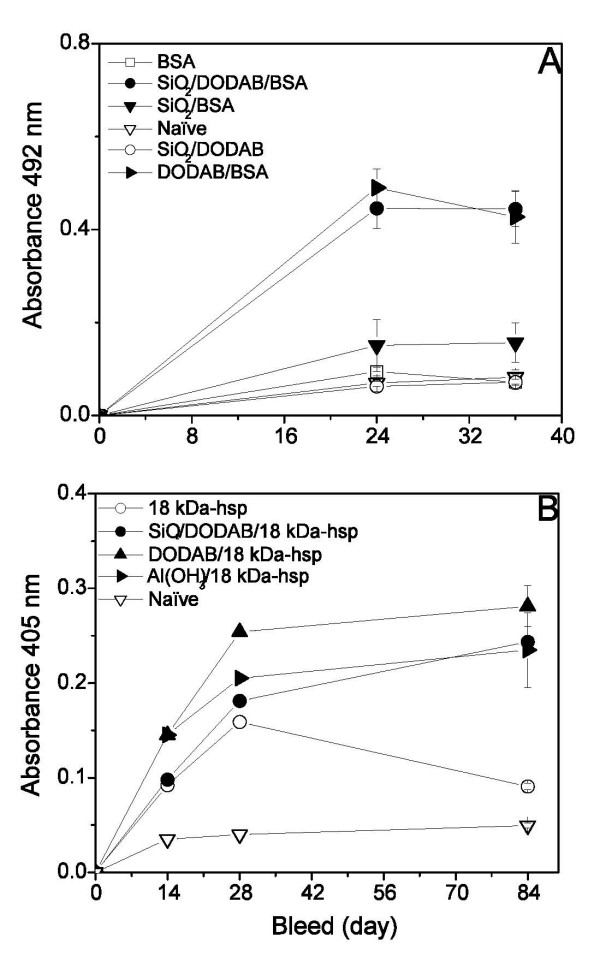
**Suitability of the silica/DODAB/antigen system to induce IgG response**. Humoral immune response to albumin and 18 kDa-hsp presented to the immune system via different systems. In A, absorbance at 492 nm as function of time (in days) after immunizing mice (n = 8/group) with 0.2 ml of 5 mM Tris/HCl (pH 7.4) solution containing: 10 μg of BSA or 0.02 mg silica or silica/DODAB (0.02 mg silica/0.01 mM DODAB) or silica/DODAB/BSA (0.02 mg silica/0.01 mM DODAB/10 μg of BSA) or DODAB/BSA (0.02 mM DODAB/10 μg of BSA). Animals were immunized on the abdomen at day zero, at two separate sites and bled through the opthalmic plexus at days 24 and 36. IgG antibodies quantification was performed by ELISA. Serum dilution was 1:50. (*), significant at *P *≤ 0.05. In B, absorbance at 405 nm as function of time (in days) after immunizing mice (n = 5/group) with: 0.3 mL of 5 mM Tris/HCl (pH 7.4) solution containing 15 μg of 18 kDa-hsp *M. leprae*, or 0.03 mg silica or silica/DODAB (0.03 mg silica/0.015 mM DODAB BF), or silica/DODAB/18 kDa-hsp *M. leprae *(0.03 mg silica/0.015 mM DODAB/15 μg of 18 kDa-hsp *M. leprae*), or Al(OH)_3_/18 kDa-hsp *M. leprae *(0.03 mg Al(OH)_3_/15 μg of 18 kDa-hsp *M. leprae*); or immunized with: 0.3 mL of 1 mM NaCl (pH 6.3) solution containing 15 μg of 18 kDa-hsp *M. leprae*/0.03 mM DODAB BF. Animals were immunized on the abdomen at two separate sites at day zero, and an identical booster dose was given on day 15. Animals were bled through the opthalmic plexus at days 14, 28 and 84. IgG antibodies quantification was performed by ELISA. Serum dilution was 1:2. Naïve mice were considered to be those injected with buffer solutions.

The immunoadjuvant effect of silica-based supported cationic bilayers was evaluated as compared to controls both from the point of view of IgG antibody production and cellular immune response as detected from delayed-type hypersensitivity (DTH) reaction [30] in mice after immunization and challenge. Silica/BSA, DODAB/BSA or silica/DODAB/BSA induce weak IgG production that can be ascribed to nonspecific antibodies given the 1:2 serum dilution employed (Figure 8A). Similarly, the 1:50 serum dilution employed to obtain IgG production elicited by silica/DODAB/18 kDa-hsp revealed a weak humoral response against this antigen (Figure 8B). Interestingly enough, there is no difference between silica/DODAB/antigen and DODAB/antigen in terms of antibody responses in the case of 18 kDa-hsp (Figure 8B). We have recently explored this to systematically study the interesting property of DODAB bilayer fragments as antigen nanocarriers by themselves. They are also able to induce good cellular immune responses at very low DODAB concentrations (unpublished results). The absorbance values in Figure 8 are very low and within the range of nonspecific responses to both antigens and might be related to the antigen preference for self-aggregation in solution instead of adsorption onto the particles as depicted from the large sizes of proteic aggregates in solution (Table 1). The immune responses to the recombinant 18 kDa-hsp from *Mycobacterium leprae *were studied in different presentations: free, copolymerized with bovine serum albumin in aggregates (18 kDa-hsp-BSA), and either surface linked to liposomes or entrapped into liposomes [41]. Measuring the antibody production of immunized genetically selected mice has compared the adjuvant effects of liposomes and proteic copolymer. Among the two liposome preparations, the strongest response was obtained with the surface-exposed antigen-liposomes[41].

Table 3 shows the % footpad swelling for control mice in comparison to those immunized with DODAB BF carrying BSA or silica/DODAB/BSA. Although BSA is a poor antigen regarding elicitation of cellular immune response due to homology of structure with mice serum albumin, the silica/DODAB/BSA particulate was so efficient that a small but significant DTH response could be detected (Table 3). On the other hand, 18 kDa-hsp was previously shown to be a potent immune modulator in conjunction with DODAB large vesicles for DTH response [29]. Therefore, in the next section, 18 kDa-hsp immobilization and presentation by silica/DODAB particles is described.

**Table 3:** Percentage footpad swelling (%fs) (delayed-type hypersensitivity reaction) to BSA or 18 kDa-hsp *M. leprae *antigens supported on DODAB-covered silica particles, or complexed with Al(OH_3_).

**Systems for sensitization**	**Ag/dose for sensitization (μg/mouse)**	**Elicitation (μg/mouse)**^**(*i*)**^			
		
		**BSA**		**18 kDa-hsp *M. leprae***	
		
		**4**	**40**	**3**	**30**
		
		***% footpad swelling ± SEM***			
Silica/DODAB^(*ii*)^	-	2 ± 2	2 ± 2	-	-
BSA	10	2 ± 2	2 ± 2	-	-
Silica/BSA^(*ii*)^	10	3 ± 2	2 ± 2	-	-
DODAB BF^(*iii*)^/BSA	10	3 ± 2	2 ± 2	-	-
Silica/DODAB/BSA^(*ii*)^	10	11 ± 2^(*iv*)^	14 ± 4^(*iv*)^	-	-
18 kDa-hsp *M. leprae*	15	-	-	4 ± 2	10 ± 2
DODAB BF^(*iii*)^/18 kDa-hsp	15			56 ± 8^*(iv), (v)*^	95 ± 16^*(iv), (v)*^
Silica/DODAB/18 kDa-hsp^(*ii*)^	15	-	-	60 ± 7^*(iv), (v)*^	89 ± 8^*(iv), (v)*^
Al(OH)_3_/18 kDa-hsp^(*ii*)^	15	-	-	18 ± 4^*(iv)*^	33 ± 1^*(iv)*^

(i) Animals were sensitized with different systems and footpad swelling was elicited on the 5^th ^day pos-immunization.

(ii) Silica at final concentration of 0.1 mg/ml; DODAB at final concentration of 0.05 mM. Al(OH)_3 _at final concentration of 0.1 mg/ml.

(iii) [DODAB] = 0.1 mM.

(iv) P < 0.05, compared to animals that were BSA or 18 kDa-hsp M. leprae-pretreated and received the same elicitation dose.

(v) P < 0.05, compared to animals that were Al(OH_3_)/18 kDa-hsp-pretreated and received the same elicitation dose.

### Silica-based cationic bilayers for induction of cellular immune response

**Figure 9:**
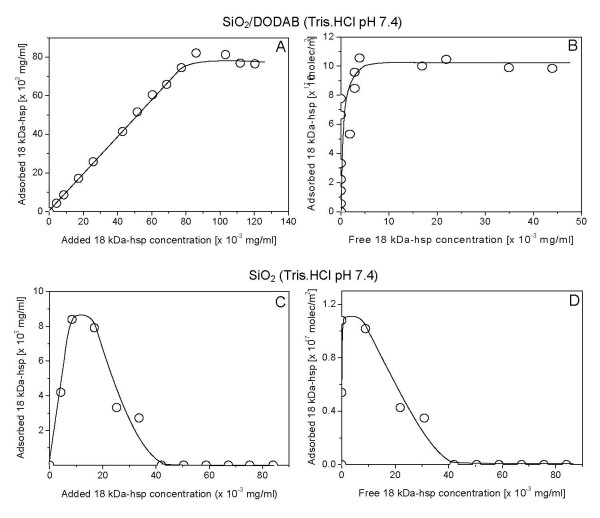
**Poor maximal adsorption of antigen on bare silica against sustained maximal adsorption onto cationized silica**. 18 kDa-hsp adsorption onto SiO_2_/DODAB particles (A-B) or bare SiO_2 _particles (C-D) in 5 mM Tris.HCl, pH 7.4. Interaction between particles and protein took place for 1 h at 25°C. Final silica and DODAB concentrations are 0.1 mg/ml and 0.05 mM, respectively. 18 kDa-hsp *M. leprae *adsorption was expressed either as adsorbed 18 kDa-hsp *M. leprae *concentration in mg/ml (A and C) or as number of 18 kDa-hsp *M. leprae *molecules adsorbed per m^2 ^silica (B and D).

The adsorption of 18 kDa-hsp onto bare silica or onto silica/DODAB particles was quantitatively evaluated over a range of 18 kDa-hsp concentrations in the mixtures (Figure 9). It was interesting to notice the distinctive shape of adsorption isotherms for both particulate systems: whereas the cationic particulate produced adsorption of high affinity leading to a plateau maximum (Figure 9A and Figure 9B), bare silica changed the adsorption curve to a typical competitive adsorption profile with protein-protein interaction inducing protein desorption from silica and leading to null adsorption over the high protein concentration regimen (Figure 9C and Figure 9D). This result emphasizes the importance of cationic character of the particulate for maintaining antigen adsorption on particles.

**Figure 10:**
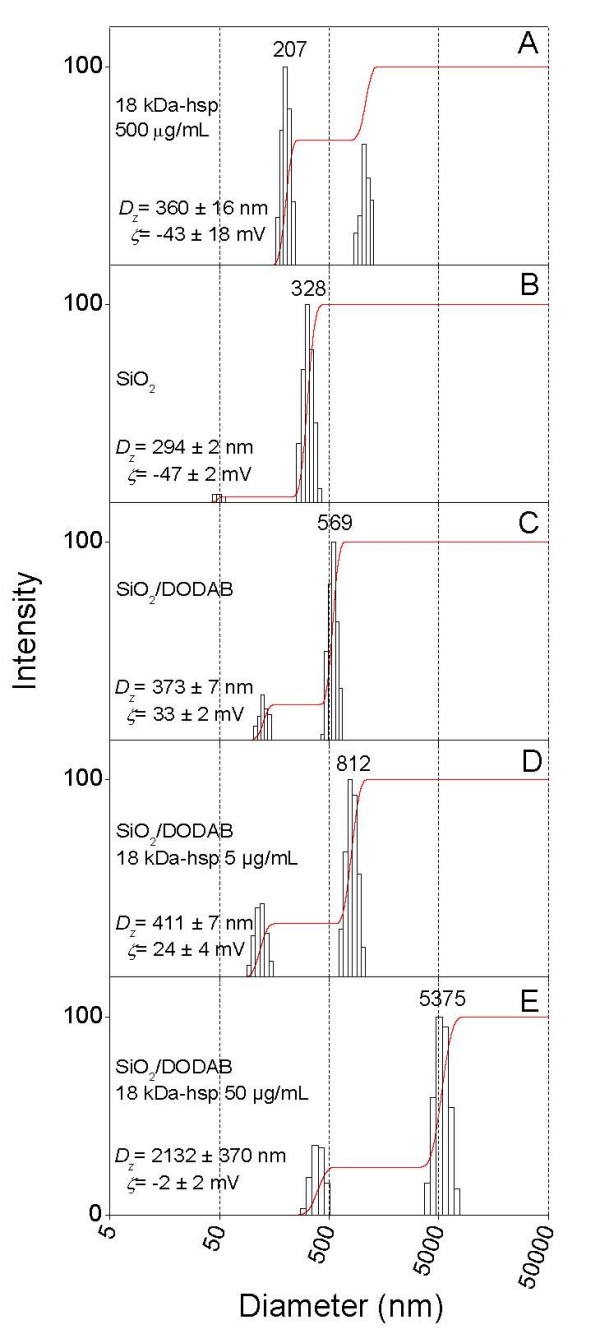
**Antigen adsorption induces silica/DODAB aggregation**. Size distribution and zeta-potential (ζ) for dispersions in 5 mM Tris.HCl, pH 7.4. In (A), 500 μg/ml 18 kDa-hsp *M. leprae*. In (B), 0.1 mg/ml silica. In (C), 0.1 mg/ml silica and 0.05 mM DODAB BF dispersion. From D to E, 0.1 mg/ml silica, 0.05 mM DODAB BF dispersion and 18 kDa-hsp *M. leprae *at 0.005 (D), and 0.05 mg/ml (E). Interaction between components in the mixtures took place over 1 h before measurements. In each subfigure, mean zeta-potential and diameter ± SE are quoted.

**Figure 11:**
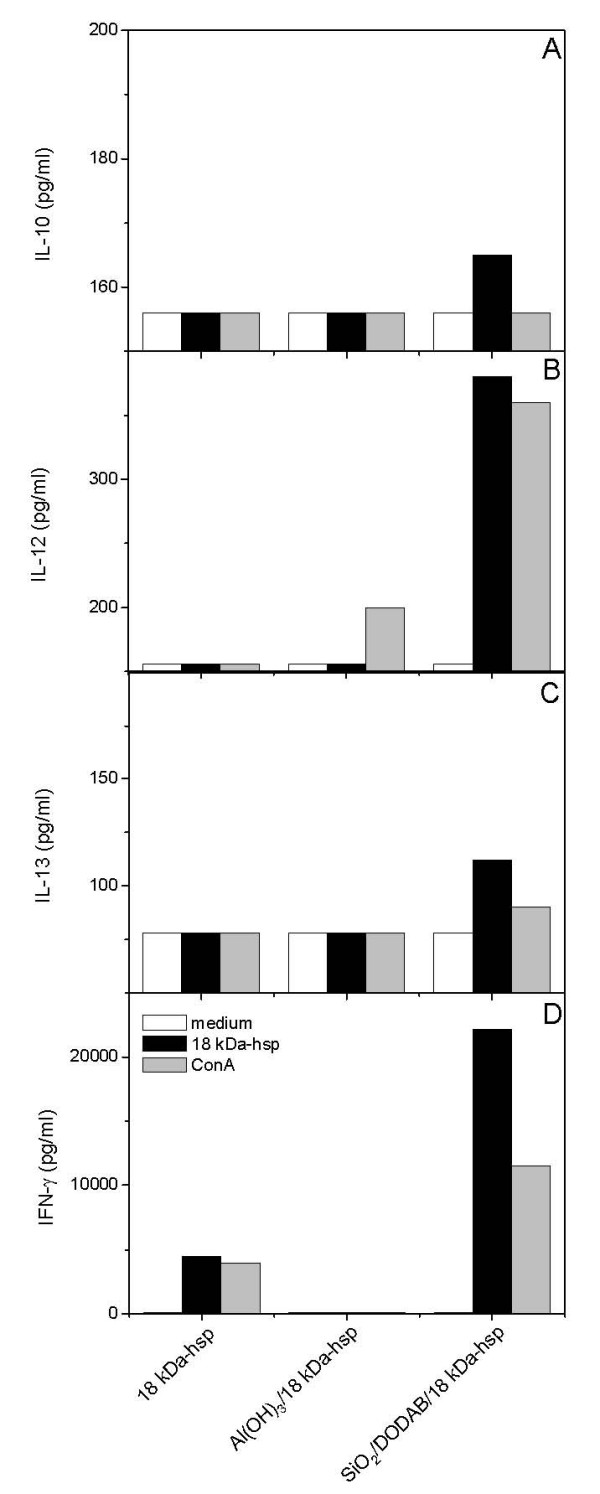
**Suitability of the silica/DODAB/antigen system to induce improved cytokines production**. Cytokine responses generated by SiO_2_/DODAB/18 kDa-hsp and Al(OH)_3_/18 kDa-hsp. Release of IL-10 (A), IL-12 (B), IL-13 (C) and IFN-γ (D) from inguinal and periaortic lymph node cells isolated from BALB/c mice immunized with 15 μg of 18 kDa-hsp *M. leprae *administered in silica/DODAB or Al(OH)_3_. Lymph node cells were isolated six days after immunization and re-stimulated in vitro with 250 μg/ml of 18 kDa-hsp or 2.5 μg/mL of ConA for 48 hours. The cytokine levels were measured by sandwich kit enzyme-linked immunosorbent assay (ELISA) as described in materials and methods. The limit of detection was 156 pg/mL for IL-10 and IL-12, and 78 pg/mL for IL-13 and IFN-γ respectively.

The 18 kDa-hsp is similar to BSA in the sense that it tends to form large aggregates in water solution (Figure 10). In Figure 10A, this aggregation is characterized at 0.5 mg/ml of 18 kDa-hsp concentration indicating negatively charged aggregates (-43 mV of zeta-potential) with 360 nm of mean hydrodynamic diameter. Adding protein at 0.005 mg/ml of final 18 kDa-hsp concentration to the silica/DODAB particles produced a positively charged particulate with 411 nm of mean diameter and zeta-potential of 24 mV (Figure 10D). Increasing final 18 kDa-hsp by ten times up to a final concentration of 0.05 mg/ml led to large colloidal instability and mean diameter (2132 nm) due to occurrence of a zeta-potential close to zero (-2 mV) (Figure 10E). Therefore, for assaying the DTH immune response, the protein concentration chosen was intermediary at 0.015 mg/ml (Table 3). The DTH response induced by silica/DODAB/18 kDa-hsp after sensitizing the mice with 0.015 mg/ml 18 kDa-hsp was excellent and higher than the one elicited by alum adjuvant under analogous conditions (Table 3). In agreement with the DTH response, cytokines production, especially INF-γ, revealed the superior character of the silica/DODAB adjuvant in comparison to alum (Figure 11). Therefore, the silica/DODAB/antigen system is prone to be used as a biocompatible cationic adjuvant for antigen presentation and vaccine design. Much larger biomimetic crystals composed of hydroxyapatite or urate with several micrometers of mean diameter and diverse crystal shapes revealed their robust effect on the expression of CD11b, MHC-class II and CD 86 on peritoneal macrophages systems [42]. The system described in this work is also expected to provide a variety of shapes since silica particles are forming aggregates from primary particles of 50 nm mean diameter. Indeed manipulation of the physico-chemical features of the particulates provides means of controlling the innate immune response.

## Conclusion

Supported cationic bilayers built on silica can effectively adsorb antigens to elicit superior immune responses *in vivo*. They can be prepared from a tiny amount of cationic and inexpensive synthetic lipid, just enough for covering silica particles with a cationic layer. The main advantage of this adjuvant system is precisely this low amount of cytotoxic cationic lipid employed in comparison to cationic liposomes usually used over a range of millimolar concentrations. Regarding physical properties, silica/DODAB particulates are less polydisperse than alum allowing better antigen presentation and eliciting superior cellular immune responses. Therefore, cationized silica is a biocompatible, inexpensive, easily prepared and possibly general immunoadjuvant for antigen presentation which displays higher colloid stability than alum and better performance regarding cellular immune responses.

## Methods

### Lipids, silica particles and antigen

Dioctadecyldimethylammonium bromide (DODAB) 99.9% pure was obtained from Sigma-Aldrich (St Louis, MO, USA). Silica (Aerosil OX-50) with a 50 nm mean diameter from transmission electron microscopy and nominally, 26 m^2^/g specific surface area was a gift from Degussa (Degussa Co.). A stock silica dispersion at 4 mg/ml was prepared in 1 mM NaCl (pH 6.3) or 5 mM Tris.HCl (pH 7.4) solutions, which provide adequate ionic strengths to assemble DODAB as a single bilayer onto particles [15]. Bovine serum albumin (BSA) was purchased from Sigma-Aldrich, and prepared as a 1 mg/ml stock solution in 1 mM NaCl (pH 6.3) or 5 mM Tris.HCl 5 mM (pH 7.4) and stored in a freezer in 1 ml aliquots for quick use. Recombinant 18 kDa-hsp *Mycobacterium leprae *protein (18 kDa-hsp) was prepared as previously described [28] and diluted in 1 mM NaCl (pH 6.3) or 5 mM Tris.HCl (pH 7.3) to obtain a stock solution at 2 mg/ml. 18 kDa-hsp concentration was determined spectrophotometrically measuring the absorbance at λ = 230 nm, using a standard curve (5 – 160 μg/ml) of 18 kDa-hsp, as previously described [43]. BSA concentration was determined by a protein microassay, based on the method of Lowry [44], using a standard curve (10 – 100 μg/ml) of BSA. Aluminium hydroxide adjuvant Al(OH_3_) was obtained from Merial do Brasil (Merial Ltda.). NaCl, Trizma base, and all other reagents were analytical grade. Water was Milli-Q quality.

### Preparation of lipidic dispersions and analytical determination of lipid concentration

Small DODAB bilayer fragments were prepared by sonication with titanium macrotip probe in 1 mM NaCl (pH 6.3) or 5 mM Tris.HCl (pH 7.4) water solution at ca. 2.0 mM DODAB (nominal potency, 80 W/15 min of sonication time) as previously described [45]. Following sonication, the solutions were centrifuged (10.000 g/15°C/40 min) to eliminate the titanium ejected from the tip. Mean size and ζ-potential for the DODAB dispersions are shown in Table 1. The DODAB concentration was determined spectrophotometrically from Orange G/DODAB solubilization in neutral micelles [46] or from halide microtitration [47]. The silica powder was routinely dispersed by sonication with a titanium tip (85 W/10 min) in 1 mM NaCl (pH 6.5) or 5 mM Tris.HCl (pH 7.5). Titanium particles ejected from the tip were allowed to pellet for 1 h before the silica dispersion was withdrawn from the supernatant for further use.

### Preparation of silica/DODAB and silica/DODAB/protein assemblies

Stock dispersions of silica particles at 4 mg/ml and stock DODAB bilayer fragments BF dispersions at 2.0 mM DODAB were dispersed either in 1 mM NaCl (pH 6.3) or in 5 mM Tris.HCl (pH 7.4) and diluted to the final desired concentration using this same solution of NaCl or Tris.HCl. First, to obtain lipid-covered silica particles, silica, at 0.1 mg/ml final concentration, and oppositely charged DODAB BF solutions ranging from 0.1 μM to 1 mM, interacted for 1 h/25°C. DODAB final co ncentration for producing the assemblies was selected as 50 μM at 0.1 mg/ml of silica since this concentration is the one required to cover each silica particle with a DODAB bilayer [15]. In fact, experimentally it is shown in Figure 1 that from this concentration cationic particles are indeed obtained. In a second experimental step, the stock BSA or 18 kDa-hsp solutions were used to obtain final protein concentrations ranging from 5 to 50 μg/ml after addition to the silica/DODAB mixture, for 1 h/25°C interaction. Thereafter, sizes, zeta-potentials, and polydispersities were determined. Considering the total surface area of 2.6 × 10^-3 ^m^2 ^the selected DODAB concentration of 0.05 mM was more than sufficient to produce bilayer-covered particles.

### Determination of average zeta-diameter and zeta-potential for particles, bilayer fragments, or mixtures of both

Particle size (mean diameter *D*_*z*_), size distribution, polydispersity and zeta-potential (ζ) in the presence or absence of silica, DODAB and BSA or 18 kDa-hsp were determined using the ZetaPlus-ZetaPotential Analyzer (Brookhaven Instruments Corporation, Holtsville, NY), which was equipped with a 677 nm laser and dynamic light scattering (PCS) at 90° for particle sizing. Mean d iameters were obtained by fitting data to log-normal size distributions which do not discriminate between one, two, or more different populations and considers always all scattering particles as belonging to one single Gaussian population. On the other hand, for the size distribution data, fitting was performed by the apparatus software using the non-negatively constrained least squares (NNLS) algorithm, which is a model independent technique allowing to achieve multimodal distributions [48]. ζ was determined from electrophoretic mobility μ in 1 mM NaCl and the Smoluchowski's equation: ζ = μη/ε, where η is the medium viscosity and ε the medium dielectric constant.

### Determination of BSA and 18 kDa-hsp adsorption isotherms

BSA adsorption isotherms on silica alone, in 1 mM NaCl (pH 6.3) or 5 mM Tris.HCl (pH 7.4), were obtained by mixing 0.05 ml of silica solution (0.4 mg/ml) with 0.15 ml of the appropriate BSA dilution in 1 mM NaCl or 5 mM Tris.HCl. For BSA adsorption onto silica/DODAB particles, prior to protein addition, 0.05 ml of silica solution was allowed to interact for 1 hour with 0.05 ml of a 0.2 mM DODAB BF dispersion; thereafter, BSA solution was added to yield a final volume of 0.2 ml. Final concentration of BSA in the assays ranged from 0 – 150 μg/ml, at a fixed concentration of 0.1 mg silica/ml and 0.05 mM DODAB BF. After 1 h silica/BSA or silica/DODAB/BSA interaction at 25°C, a clear supernatant was obtained by centrifugation at 15,000 rpm for 1.5 h. The concentration of protein in the supernatant was determined by Lowry microassay using a standard curve prepared from 10 – 100 μg/ml BSA [38]. The method is sensitive over a protein concentration range of 0.005–0.100 mg/ml. A microplate reader equipped with a 655 nm filter (Ultramark, Model 550 Bio-Rad, Hercules, CA, USA) was used for absorbance measurement.

18 kDa-hsp adsorption isotherms on silica alone, in 5 mM Tris.HCl (pH 7.4), were obtained by mixing 0.025 ml of stock silica dispersion (4 mg/ml) with 0.975 ml of the appropriate 18 kDa-hsp dilution in 5 mM Tris.HCl. For 18 kDa-hsp adsorption onto silica/DODAB particles, prior to protein addition, 0.025 ml of stock silica dispersion was allowed to interact for 1 hour with 0.025 ml of a 2 mM DODAB BF dispersion; thereafter, 18 kDa-hsp solution was added to yield a final volume of 1 ml. Final concentration of 18 kDa-hsp in the assays ranged from 0 – 120 μg/ml, at a fixed concentration of 0.1 mg silica/ml and 0.05 mM DODAB BF. After 1 h silica/18 kDa-hsp or silica/DODAB/18 kDa-hsp interaction at 25°C, a clear supernatant was obtained by centrifugation at 15,000 rpm for 1.5 h. The concentration of protein in the supernatant was determined spectrophotometrically by measuring the absorbance at λ = 230 nm, using a standard curve (5 – 160 μg/ml) of 18 kDa-hsp, as previously described [37]. A spectrophotometer Hitachi U-2000 was used for absorbance measurement.

For both proteins, the amount of adsorbed protein was determined by the difference between the total protein added and the amount of protein recovered in the supernatant.

Adsorption was expressed as the number of molecules adsorbed per square meter silica. Curves were fitted using cubic polynomial regression. Wherever possible, the Langmuir model was employed for isotherms linearization and determination of adsorption constants such as affinity constant (K, in M^-1^) and maximal adsorption (in number of molecules per m^2 ^silica) [49].

### Subcutaneous immunization, assay for delayed-type hypersensitivity (DTH) and antigen-specific ELISA

BALB/c female mice 8–12-week old were purchased from the University of São Paulo, São Paulo, Brazil. Six groups of 8 – 10 female mice were challenged subcutaneously (s. c.) in the abdomen at two separate sites. Total volume injected in each site was 0.1 or 0.15 mL for BSA or 18 kDa-hsp, respectively. The dispersion injected contained either: (a) 10 μg BSA or 15 μg 18 kDa-hsp in 5 mM TrisHCl (pH 7.4); or (b) 10 μg BSA or 15 μg 18 kDa-hsp in 1 mM NaCl and 0.1 mM DODAB; or (c) 10 μg BSA or 15 μg 18 kDa-hsp in 0.1 mg silica/mL, 5 mM Tris.HCl; or (d) 10 μg BSA or 15 μg 18 kDa-hsp in silica/DODAB (0.1 mg mL^-1^/0.05 mM) in 5 mM Tris.HCl; or (e) 0.1 mg silica/mL in 5 mM Tris.HCl. For DTH evaluation, the footpad swelling test was carried out essentially as previously described [9,26,27]. On the fifth day post subcutaneous immunization, pre-immunized mice with BSA, 18 kDa-hsp, DODAB/BSA, DODAB/18 kDa-hsp, silica/BSA, silica/18 kDa-hsp, silica/DODAB/BSA, silica/DODAB/18 kDa-hsp or silica alone, were challenged in the left-hind footpad with a total elicitation dose of 4 or 40 μg BSA or 3 or 30 μg 18 kDa-hsp in 50 μL Tris.HCl 5 mM, respectively. Footpad swelling was measured 24 h later with a Mitutoyo engineering micrometer. Depending on the age of the animals, the thickness of uninjected hind footpad varied from 1.60 to 1.70 mm. Percentage footpad swelling (%fs) is calculated according to the formula below with results expressed as %fs ± standard error of the mean (SEM).

%fs = 100 [(left hind footpad thickness) - (right hind footpad thickness)]/(mean thickness uninjected left hind footpad).

For evaluation of humoral immune response, the same groups previously immunized and challenged with BSA above were bled through the ophthalmic plexus in days 24 and 36, after immunization. The sera obtained were analyzed by ELISA. Each well of 96-well ELISA polystyrene high binding plates (Costar Corning Inc., Cambridge, Mass.) was coated with 100 μL of BSA (final concentration of 0.05 μg/well) in 0.5 M carbonate-bicarbonate buffer (pH 9.6) for 18 h in an humidified chamber at 4°C. The wells were blocked for 1 h with 5% milk in PBS containing 0.05% Tween 20 (PBS/T), and then incubated for one hour with serum samples diluted 1:50 or 1:200, for specific IgG antibody quantitation. In each well, 100 μL of goat anti-mouse IgG peroxidase-conjugate (Sigma) diluted 1:3000 was added and plates were incubated for 1 h. After each incubation step, the plates were washed using an automatic washer, with four cycles of PBS/T. Ortho-phenylenediamine (1 mg/mL) (Sigma) and H_2_O_2 _(1 μL/mL) diluted in 0.2 M citrate buffer (pH 5.0) were added (in the dark) as chromogenic substrate and plates were incubated for 10 min. The reactions were stopped by adding 100 μL of 2 M H_2_SO_4_. Color intensity was quantified using an ELISA plate reader (Diagnostics Pasteur, Strassburg-Schiltigheim, France) at 492 nm. All incubations were carried out at 37°C. Antibody titers remained below 1/100 dilution, meaning that the dilution is not high enough to discriminate specific and non specific antibodies against BSA. Serum titration included a serum from naïve mice and a serum from mice immunised with a non relevant antigen towards induction of humoral immune response, namely, hsp-18 kDa protein itself.

For evaluation of humoral immune response against 18 kDa-hsp, the same groups previously immunized and challenged with 18 kDa-hsp above were bled through the ophthalmic plexus in days 14, 28, and 84 after immunization. The sera obtained were analyzed by ELISA. Each well of 96-well ELISA polystyrene maxisorpt plates (Maxisorp, Nunc) was coated with 100 μL of 18 kDa-hsp (final concentration of 40 μg/well) in 0.5 M carbonate-bicarbonate buffer (pH 9.6) for 18 h in an humidified chamber at 4°C. The wells were blocked for 1 h with 5% milk in PBS containing 0.05% Tween 20 (PBS/T), and then incubated for one hour with serum samples diluted 1:2, for specific IgG antibody quantitation. In each well, 100 μL of goat anti-mouse IgG peroxidase-conjugate (Sigma) diluted 1:1000 was added and plates were incubated for 1 h. After each incubation step, the plates were washed using an automatic washer, with four cycles of PBS/T. Ortho-phenylenediamine (1 mg/mL) (Sigma) and H_2_O_2 _(1 μL/mL) diluted in 0.2 M citrate buffer (pH 5.0) were added (in the dark) as chromogenic substrate and plates were incubated for 10 min. The reactions were stopped by adding 100 μL of 2 M H_2_SO_4_. Color intensity was quantified using an ELISA plate reader A microplate reader equipped with a 405 nm filter (Ultramark, Model 550 Bio-Rad, Hercules, CA, USA) was used for absorbance measurement. All incubations were carried out at 37°C.

### Cell culture and cytokine analysis

Six days after immunization, cell suspensions from inguinal and periaortic lymph nodes (LN) of 5 mice were prepared in RPMI 1640 (GIBCO) supplemented with 10 mM HEPES, 50 μM 2-Mercaptoethanol, 216 mg L-glutamine/L and 5 % FCS (GIBCO). The cell suspensions (8 × 10^6 ^cells) were distributed into tissue culture 24-wells plates (Costar) and incubated with medium containing 250 μg/mL of 18 kDa-hsp *M. leprae *in a humidified CO_2 _incubator for 48 hours. Wells containing medium only or 2.5 μg/mL of ConA (Sigma Aldrich) were included in all experiments as negative and positive controls, respectively. After this time, the plates were centrifuged for 8 minutes at 500 × g and the supernatants collected for cytokine content. The levels of cytokines (IL-10, IL12, IL-13 and IFN-γ) in the supernatants were assayed by sandwich kit enzyme-linked immunosorbent assay (ELISA), using the following monoclonal antibodies: MAB417 and biotinylated-BAF417 for IL-10; MAB419 and biotinylated-BAF419 for IL-12; MAB413 and biotinylated-BAF413 for IL-13; and XMG 1.2 and biotinylated-AN18 for IFN-γ, according to the manufacturer's suggestion (R&D Systems – Minneapolis, MN). Binding of the biotinylated antibodies was determined using the streptavidin-peroxidase conjugate (Sigma) and TMB (3,3',5,5'-Tetramethylbenzidine; Sigma) solution in citrate buffer plus Hydrogen peroxide. The plates were read (450 nm) in an automated ELISA reader (Dynatech MR5000). Samples were quantified by comparison with standard curves of purified recombinant cytokines and the values expressed as ng/mL. The limit of detection was 156 pg/mL for IL-10 and IL-12, and 78 pg/mL for IL-13 and IFN-γ respectively.

### Statistical analysis

ANOVA one-way multiple comparison tests and the Kruskal-Wallis non-parametric test were used when needed. A *P *value of ≤ 0.05 was considered significant.

## Competing interests

The authors declare that they have no competing interests.

## Authors' contributions

NL did most experiments and data analysis in the laboratory, MRAS obtained adsorption isotherms and did some sizing and zeta-potential determinations, EFM performed cytokines experiments and analysis, MHBC scaled-up production of the 18 kDa-hsp recombinant protein from *M. leprae *and provided this protein for the experiments, AMCR coordinated the experiments, provided important advice for the experiments and financial support. All authors read and approved the final manuscript.

## References

[B1] Al-Jamal WT, Kostarelos K (2007). Liposome-nanoparticle hybrids for multimodal diagnostic and therapeutic applications. Nanomedicine.

[B2] Troutier AL, Ladavière C (2007). An overview of lipid membrane supported by colloidal particles. Adv Colloid Interface Sci.

[B3] Carmona-Ribeiro AM (2007). Biomimetic particles in drug and vaccine delivery. J Liposome Res.

[B4] Petri DFS, Carmona-Ribeiro AM, Nalwa HS (2007). Biomimetic particles. Polymeric Nanostructures and Their Applications.

[B5] Sicchierolli SM, Carmona-Ribeiro AM (1996). Biomolecular recognition on phospholipid-covered polystyrene microspheres. J Phys Chem.

[B6] Moura SP, Carmona-Ribeiro AM (2006). Biomimetic particles for isolation and reconstitution of receptor function. Cell Biochem Biophys.

[B7] Pacheco LF, Carmona-Ribeiro AM (2003). Effects of synthetic lipids on solubilization and colloid stability of hydrophobic drugs. J Colloid Interface Sci.

[B8] Lincopan N, Carmona-Ribeiro AM (2006). Lipid-covered drug particles: combined action of dioctadecyldimethylammonium bromide and amphotericin B or miconazole. J Antimicrob Chemother.

[B9] Lincopan N, Espíndola NM, Vaz AJ, Carmona-Ribeiro AM (2007). Cationic supported lipid bilayers for antigen presentation. Int J Pharm.

[B10] Lincopan N, Rosa H, Carmona-Ribeiro AM (2006). Biomimetic particles. Macromol Symp.

[B11] Blau S, Jubeh TT, Haupt SM, Rubinstein A (2000). Drug targeting by surface cationization. Crit Rev Ther Drug Carrier Syst.

[B12] Pereira EMA, Vieira DB, Carmona-Ribeiro AM (2004). Cationic bilayers on polymeric particles: effect of low NaCl concentration on surface coverage. J Phys Chem B.

[B13] Rapuano R, Carmona-Ribeiro AM (1997). Physical adsorption of bilayer membranes on silica. J Colloid Interface Sci.

[B14] Rapuano R, Carmona-Ribeiro AM (2000). Supported bilayers on sílica. J Colloid Interface Sci.

[B15] Moura SP, Carmona-Ribeiro AM (2003). Cationic bilayer fragments on silica at low ionic strength: competitive adsorption and colloid stability. Langmuir.

[B16] Carter DC, Ho JX (1994). Structure of serum albumin. Adv Protein Chem.

[B17] Malmsten M (2003). Biopolymers at interfaces.

[B18] Gray JJ (2004). The interaction of proteins with solid surfaces. Curr Opin Struct Biol.

[B19] Bodzon-Kulakowska A, Bierczynska-Krzysik A, Dylag T, Drabik A, Suder P, Noga M, Jarzebinska J, Silberring J (2007). Methods for samples preparation in proteomic research. J Chromatogr B Analyt Technol Biomed Life Sci.

[B20] Efimova YM, Haemers S, Wierczinski B, Norde W, van Well AA (2007). Stability of globular proteins in H_2_O and D_2_O. Biopolymers.

[B21] Norde W, Buijs J, Lyklema H, Lyklema J (2005). Adsorption of globular proteins. Fundamentals of interface and colloid science.

[B22] Veen M Van der, Stuart MC, Norde W (2007). Spreading of proteins and its effect on adsorption and desorption kinetics. Colloids Surf B: Biointerfaces.

[B23] Derand H, Malmsten M (2003). Protein interfacial behavior in microfabricated analysis systems and microarrays. Surfactant Sci Ser.

[B24] Lincopan N, Carmona-Ribeiro AM (2009). Protein assembly onto cationic supported bilayers. J Nanoscience Nanotechnol.

[B25] Carvalho LA, Carmona-Ribeiro AM (1998). Interactions between cationic vesicles and serum proteins. Langmuir.

[B26] Mustafa AS, Lundin KE, Oftung F (1993). Human T cells recognize mycobacterial heat shock proteins in the context of multiple HLA-DR molecules: studies with healthy subjects vaccinated with *Mycobacterium bovis *BCG and *Mycobacterium leprae*. Infect Immun.

[B27] Pinho JR, Cardi BA, Andrade HF, Barr PJ, Bathurst IC, Vicente EJ, Schenberg AC (1995). Immunogenic properties of the *Mycobacterium leprae *recombinant 18-kDa antigen purified from *Saccharomyces cerevisiae*; enhancement of delayed-type hypersensitivity after gamma-irradiation. Int J Lepr Other Mycobact Dis.

[B28] Costa MHB, Ueda C, Sato RA, Liberman C, Raw I (1995). Procedures for scaling up the recombinant 18 kDa-hsp lepra protein production. Biotechnol Tech.

[B29] Tsuruta LR, Quintilio W, Costa MH, Carmona-Ribeiro AM (1997). Interactions between cationic liposomes and an antigenic protein: the physical chemistry of the immunoadjuvant action. J Lipid Res.

[B30] Snippe H, Belder M, Willers JM (1977). Dimethyl diotadecyl ammonium bromide as adjuvant for delayed hypersensitivity in mice. Immunology.

[B31] Klinguer C, Beck A, De-Lys P, Bussat MC, Blaecke A, Derouet F, Bonnefoy JY, Nguyen TN, Corvaïa N, Velin D (2001). Lipophilic quaternary ammonium salt acts as a mucosal adjuvant when co-administered by the nasal route with vaccine antigens. Vaccine.

[B32] Davidsen J, Rosenkrands I, Christensen D, Vangala A, Kirby D, Perrie Y, Agger EM, Andersen P (2005). Characterization of cationic liposomes based on dimethyldioctadecylammonium and synthetic cord factor from M. tuberculosis (trehalose 6,6'-dibehenate)-a novel adjuvant inducing both strong CMI and antibody responses. Biochim Biophys Acta.

[B33] Mohammed AR, Bramwell VW, Coombes AGA, Perrie Y (2006). Lyophilisation and sterilisation of liposomal vaccines to produce stable and sterile products. Methods.

[B34] Vangala A, Bramwell VW, McNeil S, Christensen D, Agger EM, Perrie Y (2007). Comparison of vesicle based antigen delivery systems for delivery of hepatitis B surface antigen. J Control Release.

[B35] Iler RK (1979). The chemistry of silica.

[B36] Parida SK, Dash S, Patel S, Mishra BK (2006). Adsorption of organic molecules on silica surface. Adv Colloid Interface Sci.

[B37] Tadros ThF, Lyklema J (1968). Adsorption of potential-determining ions at the silica-aqueous electrolyte interface and the role of some cations. J Electroanal Chem.

[B38] Moura SP, Carmona-Ribeiro AM (2007). Adsorption behavior of DODAB/DPPC vesicles on silica. J Colloid Interface Sci.

[B39] Callahan PM, Shorter AL, Hem SL (1991). The importance of surface charge in the optimization of antigen-adjuvant interactions. Pharm Res.

[B40] Clausi AL, Merkley SA, Carpenter JF, Randolph TW (2008). Inhibition of aggregation of aluminum hydroxide adjuvant during freezing and drying. J Pharm Sci.

[B41] Costa MHB, Sant'Anna OA, de Araujo PS, Sato RA, Quintilio W, Silva LV, Matos CR, Raw I (1998). Conformational stability and antibody response to the 18 kDa heat-shock protein formulated into different vehicles. Appl Biochem Biotechnol.

[B42] Ramesh M, Turner LF, Yadav R, Rajan TV, Vella AT, Kuhn LT (2007). Effects of the physico-chemical nature of two biomimetic crystals on the innate immune response. Int Immunopharmacol.

[B43] Costa MHB, Sato RA, Silveira A, Barratt G, Fattal E, Quintilio W (1997). Microquantification of proteins with low chromophore contents. Biotech Tech.

[B44] Lowry OH, Rosebrough NJ, Farr AL, Randall R (1951). Protein measurement with the Folin phenol reagent. J Biol Chem.

[B45] Vieira DB, Carmona-Ribeiro AM (2001). Synthetic bilayer fragments for solubilization of. amphotericin B. J Colloid Interface Sci.

[B46] Stelmo M, Chaimovich H, Cuccovia IM (1987). Quantitative determination of alkylammonium amphiphiles using neutral micelles. J Colloid Interface Sci.

[B47] Schales O, Schales SS (1941). A simple and accurate method for the determination of chloride in biological fluids. J Biol Chem.

[B48] Grabowski E, Morrison I, Dahneke B (1983). Particle size distribution from analysis of quasi-elastic light scattering data. Measurements of Suspended Particles by Quasi-Elastic Light Scattering.

[B49] Carmona-Ribeiro AM (2001). Bilayer vesicles and liposomes as interface agents. Chem Soc Rev.

